# The Oxygen Partial Pressure Dependence of Space Charges at SrTiO_3_|Mixed Ionic Electronic Conducting Oxide Heterojunctions

**DOI:** 10.1002/smtd.202500728

**Published:** 2025-06-11

**Authors:** Claudia Steinbach, Alexander Schmid, Tobias M. Huber, Juergen Fleig

**Affiliations:** ^1^ TU Wien, Institute of Chemical Technologies and Analytics Vienna 1060 Austria

**Keywords:** electrochemical impedance spectroscopy, mixed ionic electronic conductors, oxygen partial pressure, space charges, SrTiO_3_

## Abstract

The electrical and electrochemical properties of mixed conducting oxides often depend on the oxygen partial pressure *p*(O_2_) and numerous studies have dealt with the *p*(O_2_) dependencies found in bulk materials. However, measurements regarding the properties of interfaces between two different mixed conducting oxides are much less common. This work investigates the interfacial space charge region in SrTiO_3_ (STO) caused by the contact with another mixed ionic and electronic conductor (MIEC), specifically La_0.6_Sr_0.4_FeO_3−δ_ (LSF), La_0.65_Sr_0.35_MnO_3−δ_ (LSM) and La_0.9_Sr_0.1_CrO_3−δ_ (LSCr). The space charge regions were investigated by means of electrochemical impedance spectroscopy at 500 °C in the broad *p*(O_2_) range of 1 bar to 10^−32^ bar. All measurable space charge potentials Δϕ show a decrease with decreasing *p*(O_2_). A model correlates the change in Δϕ with the strongly *p*(O_2_) dependent defect concentrations of the MIECs. The measured partial pressure dependencies of Δϕ can be fully attributed to the different *p*(O_2_) dependencies of the respective bulk Fermi levels. The suggested model is broadly applicable to MIEC|MIEC interfaces in general and can also be used to predict transitions of space charges from hole depleted to electron depleted layers.

## Graphical abstract

Impedance measurements of La_0.6_Sr_0.4_FeO_3−δ_, La_0.65_Sr_0.35_MnO_3−δ_ and La_0.9_Sr_0.1_CrO_3−δ_ on top of STO (100) single crystals are preformed in a broad *p*(O_2_) range between 1 and 10^−32^ bar. The space charge regions in STO are characterized in dependence of the varying *p*(O_2_) and a model is introduced to describe and predict *p*(O_2_) dependent trends of the space charge potential.

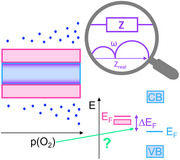

## 1 Introduction

Mixed ionic and electronic conductors (MIECs) constitute a class of material with multiple applications in energy and material science: Oxides with comparatively high electronic conductivity, such as (La, Sr)FeO_3−δ_ (LSF) or (La, Sr)CoO_3−δ_ (LSC), are frequently used as cathodes in solid oxide fuel cells.^[^ ^1^, ^2^, ^3^, ^4^ ^]^ Mixed Li‐ion and electron conductors are commonly used electrode materials in Li‐ion batteries.^[^ ^5^, ^6^, ^7^, ^8^ ^]^ Mixed conducting oxides that exhibit a variable oxygen stoichiometry, such as LSF, LSC, or (La, Sr)(Cr, Mn)O_3−δ_ (LSCrMn), are considered for electrodes in oxygen ion batteries.^[^ ^9^, ^10^ ^]^ Another group of mixed conductors is comprised of large band gap perovskite oxides, such as BaTiO_3_ or SrTiO_3_ (STO). Their properties can be tailored from insulating in capacitors,^[^ ^11^, ^12^, ^13^ ^]^ to conducting for e.g. memristors or positive temperature coefficient resistors.^[^ ^14^, ^15^, ^16^, ^17^ ^]^ Given their relevance in material science, MIEC bulk properties are frequently studied and for numerous oxides, defect chemical models exist to describe the bulk defect concentrations in a broad *p*(O_2_) range, e.g., for STO^[^ ^18^, ^19^, ^20^ ^]^ or LSF.^[^ ^21^, ^22^ ^]^ Moreover, many studies dealt with the kinetics of oxygen exchange at the surface of such MIECs.^[^ ^23^, ^24^, ^25^, ^26^, ^27^, ^28^ ^]^

It is known that also interfacial charge redistribution and formation of space charge layers may become important in MIECs. Such space charge effects have been studied, for example, for MIEC grain boundaries, ionic conductor|insulator interfaces and MIEC surfaces.^[^ ^29^, ^30^, ^31^, ^32^, ^33^ ^]^ Given that MIECs exhibit both ionic and electronic charge carriers, a redistribution of ionic as well as electronic defects takes place and can lead to many different features including grain boundary properties being blocking, e.g. found in SrTiO_3_,^[^ ^34^, ^35^, ^36^, ^37^ ^]^ or accelerating.^[^ ^38^, ^39^, ^40^ ^]^ Moreover, the contributions of different space charge effects to charge storage in solid oxide batteries have been considered.^[^ ^41^ ^]^ However, studies on the space charge region between two MIECs are rather scarce.

In a previous work^[^ ^42^ ^]^ we investigated the space charges of several STO|MIEC interfaces (MIEC = (La, Sr)FeO_3−δ_, (La, Sr)CrO_3−δ_, etc.) at high *p*(O_2_) and proposed a model to describe and predict space charge potentials. There, it is discussed how the different charge carriers contribute to the space charge region and how this is related to the thermodynamic data of oxygen vacancy formation. This model serves as a general description of how space charges at interfaces between two MIECs are formed. However, given that the charge carrier concentrations in such MIECs are strongly *p*(O_2_) dependent, one would expect *p*(O_2_) dependencies of the charge carrier concentrations also in the space charge region.

In this work, we present impedance measurements taken of heterojunctions between nominally undoped STO single crystals and hole conducting LSF, (La, Sr)MnO_3−δ_ (LSM) and (La, Sr)CrO_3−δ_ (LSCr) at 500 °C in the broad *p*(O_2_) range between 1 and 10^−32^ bar. From the measured impedance data, *p*(O_2_) dependent space charge potentials are derived for each heterojunction. We extend our previously introduced model to predict the *p*(O_2_) dependence of space charges between mixed conductors in equilibrium with the gas phase. The excellent agreement between this model and the experimental data justifies a further extension of the model by including transitions from hole to electron conductors. This further illustrates that the given approach is indeed a powerful tool to relate space charge effects to defect chemical models, i.e., Brouwer diagrams.

## 2 Experimental Section

### 2.1 Sample preparation

**Figure 1:**
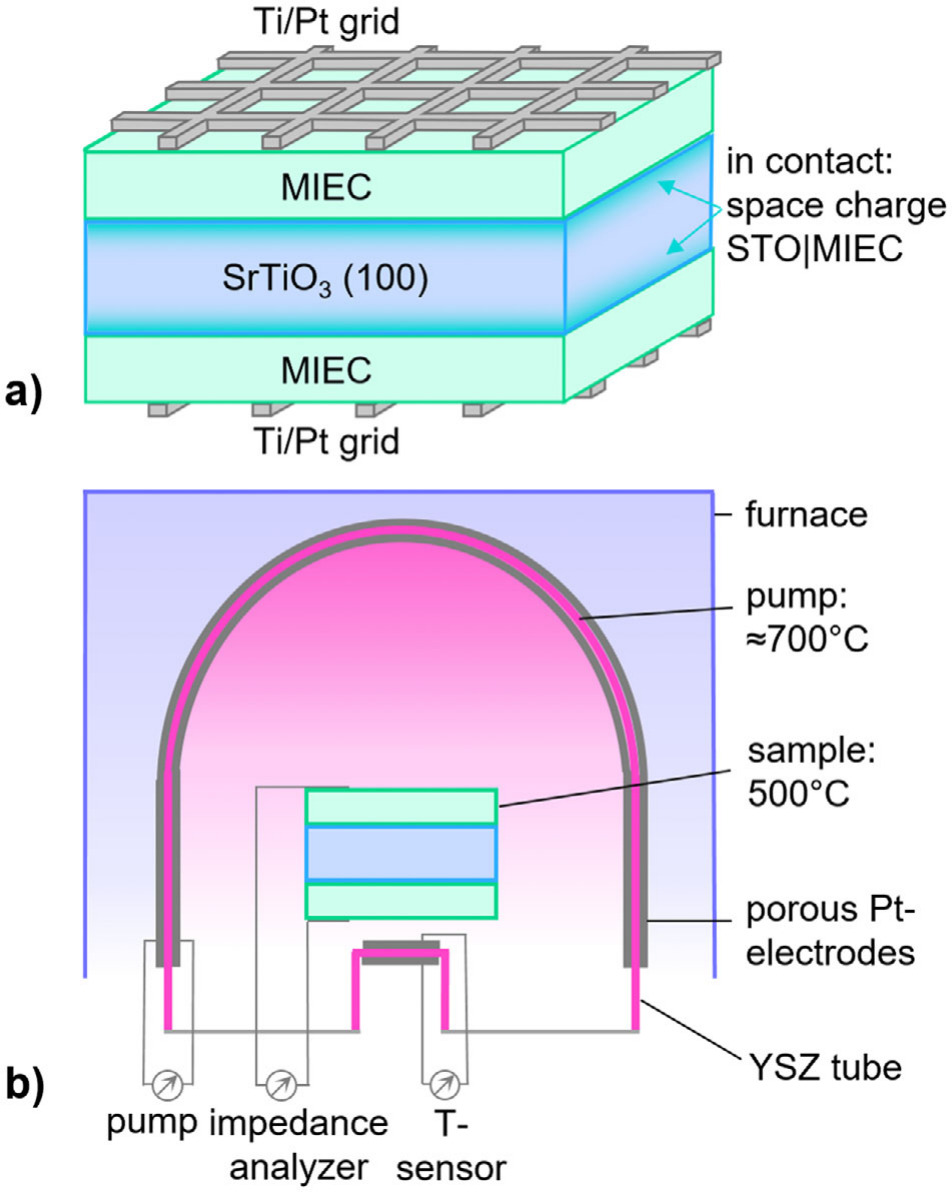
a) Schematic illustration of the used sample geometry. Onto a STO (100) single crystal MIEC thin films and Ti/Pt current collector grids were deposited symmetrically. b) Sketch of the oxygen pumping measurement setup, which consisted of a YSZ tube, coated from both sides with porous Pt and sealed at the bottom. Sample connection was established using Pt wires.

Undoped STO (100) single crystal substrates (5x5x0.5 mm^3^, Crystec (Germany), both sides polished) were used and cleaned with ethanol. By means of pulsed laser deposition, thin films of three different MIEC materials were symmetrically deposited onto the substrates. The materials and PLD parameters are listed in **Table** **1**. Each film was deposited at a target‐substrate distance of 6 cm, a pulse frequency of 5 Hz, 4500 pulses and a laser fluence of 1 J cm^−2^ which results in a film thickness of roughly 50 nm. Using photolithography and DC‐magnetron sputtering (Baltec MED020), Pt current collector grids with a strip width of 15 µm, 35 µm mesh distances and a thickness of 100 nm were deposited onto the MIEC thin films. A Ti layer of 5 nm thickness was applied between each MIEC thin film and the Pt grid to act as adhesion layer for the Pt thin film. The used sample geometry is sketched in **Figure** **1**a.

**Table 1:** PLD parameters for the deposition of the three MIEC thin films used in this study.

MIEC thin film	p(O_2_)	temperature
La_0.6_Sr_0.4_FeO_3‐δ_ (LSF)	4 · 10^−2^ mbar	600 °C
La_0.65_Sr_0.35_MnO_3‐δ_ (LSM)	4 · 10^−2^ mbar	600 °C
La_0.9_Sr_0.1_CrO_3‐δ_ (LSCr)	1.5 · 10^−2^ mbar	700 °C

### 2.2 Measurement Setup and Electrochemical Characterization

An oxygen pumping setup^[^ ^43^, ^44^ ^]^ by Huber Scientific (Austria), as shown schematically in Figure 1b, was used to establish and control the oxygen partial pressure inside the sample chamber. The oxygen pump consisted of a sealed YSZ tube that was brushed with porous Pt paste on the inside and outside. Both Pt areas were contacted through Pt wires. By applying a bias voltage to the Pt|YSZ|Pt cell, oxygen was pumped into and out of the measurement setup. Also sample connection was established through Pt wires. The measurement setup was placed in a furnace that was set to 725 °C, to ensure a sufficiently high temperature of the oxygen pump for proper oxygen transport kinetics. For measurements at higher oxygen partial pressures, the measurement setup was flushed with pure O_2_ gas before pumping, while for the lower *p*(O_2_) range 1% O_2_ gas was used for faster and more efficient pumping. The oxygen pumping and impedance measurements were conducted in a static gas atmosphere, i.e., a closed setup. The oxygen partial pressure at the sample was determined by using the STO single crystal as conductivity based O_2_ sensor, see results. The temperature gradient in the furnace enabled a temperature difference between the O_2_ pumping zone and the sample. The sample itself was held at 500 °C. This sample temperature was determined by the use of a thermocouple and further confirmed by measuring the conductivity of the STO single crystal and comparing it with the known temperature dependent conductivity values of such single crystals.^[^ ^18^ ^]^ (Please note that the actual distance between the top of the oxygen pumping setup (at 700 °C) and the sample (at 500 °C) amounts to ca. 10 cm. Due to the very thin sample thickness of 500 µm, a significant thermal gradient across the sample is not expected.)

Impedance measurements were carried out using a Novocontrol Technologies Alpha‐A High Performance Frequency Analyzer and a 4 Wire Impedance Test Interface. The investigated frequencies ranged from 1 MHz to 30 mHz with an applied AC_RMS_ voltage of 20 mV. To ensure equilibration of the sample after stepwise *p*(O_2_) changes, the resistance of the sample was monitored. A change of less than 1.5% in the sample resistance of three consecutive impedance spectra was deemed sufficiently equilibrated. Based on the gained knowledge of equilibration times, partial pressure changes were made continuously in a second approach, but sufficiently slow to warrant that sample equilibration and recording of one impedance spectrum were much faster than the partial pressure change. Therefore each impedance spectrum essentially represents a sample equilibrated to one partial pressure. The impedance spectra of these equilibrium states were analyzed using the software ZView (Scribner). The fit parameters were then used to calculate further parameters, such as the space charge potential and the oxygen partial pressure.

### 2.3 Statistical Analysis

For each investigated MIEC (LSF, LSM, and LSCr), one sample was characterized through impedance measurements using a Novocontrol Technologies Alpha‐A High Performance Frequency Analyzer and a 4 Wire Impedance Test Interface. Previous investigations were performed on several samples in the high *p*(O_2_) range.^[^ ^42^ ^]^ Those showed that sample scattering for several nominally identical samples lead to space charge potentials with standard deviations of 20 mV. The measured impedance data was fitted using ZView (Scribner). For all samples, typical fit errors for the resistance of the STO bulk *R*_STO_ and the corresponding capacitance *C*_STO_ are < 1% throughout the entire *p*(O_2_) range. For LSF, fitting errors of the space charge resistance are around 1.5% at high *p*(O_2_) and increase up to 30% in the mid *p*(O_2_) range, while the fitting errors for the capacitance of the space charge feature amount to 7% at high *p*(O_2_) and increase up to 40% at intermediate oxygen partial pressures. For LSM, typical fitting errors of the space charge resistance are 1% and 5% for the space charge capacitance, both at high *p*(O_2_). Toward intermediate *p*(O_2_), the fitting errors increase up to 15% for the space charge resistance and up to 25% for the space charge capacitance. For LSCr, typical fitting errors at high *p*(O_2_) are < 1% for the space charge resistance and ⩽1.5% for the space charge capacitance. Those fitting errors increase at intermediate *p*(O_2_) up to 15% and 25% for the space charge resistance and capacitance, respectively. Space charge potentials were calculated using fitted resistances from impedance spectroscopy. The fitting errors of the resistances translate to an error in Δϕ in the 1% and ⩽5% range for high and intermediate *p*(O_2_), respectively.

For all investigated samples, fitting errors for the resistance of the low frequency arc using a parallel RC element yield ⩽2% at high oxygen partial pressures and increase up to 5% in the intermediate *p*(O_2_) range. Toward very low *p*(O_2_) the fitting errors decrease again down to the same error values found for high *p*(O_2_). The fitting errors for the capacitance of the low frequency feature are ⩽5% in the high *p*(O_2_) range, increase up to 7% in the mid *p*(O_2_) range and decrease again to ⩽5% at very low *p*(O_2_). Fitting errors of the low frequency feature using a parallel R‐CPE element amount to ⩽3%, 6% and ⩽2% for the resistance at high, intermediate and low *p*(O_2_), respectively. The fitting errors for the corresponding capacitance yield ⩽2.5%, 6% and ⩽2% at high, intermediate and low *p*(O_2_), respectively. The error bars plotted throughout this work in the mid *p*(O_2_) range refer to the error of the *p*(O_2_) determination in dependence of the method used, i.e. the oxygen partial pressure deduced from the parallel RC element and the *p*(O_2_) determined through the parallel R‐CPE element, see below.

Data used for the Brouwer diagrams of LSF and STO was taken from literature.^[^ ^18^, ^21^ ^]^

## 3 Results and Discussion

### 3.1 Impedance Spectra and Their Interpretation

**Figure 2:**
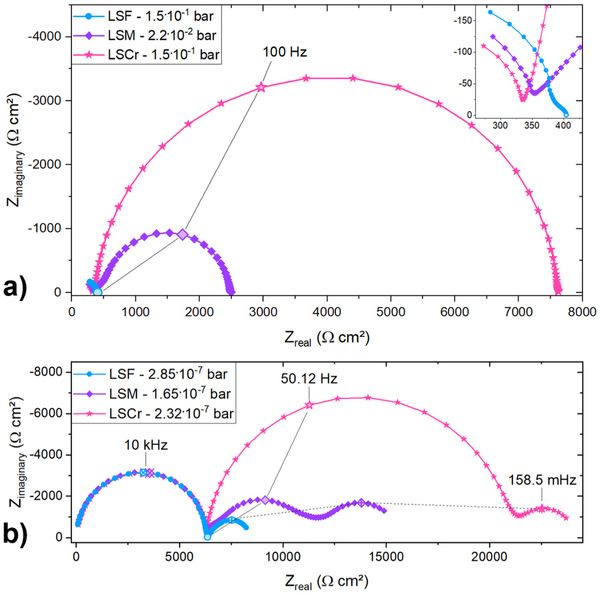
Typical Nyquist plots of LSF, LSM and LSCr on STO at 500 °C around 10^−1^ to 10^−2^ bar (a) and around 2 · 10^−7^ bar (b).

**Figure 3:**
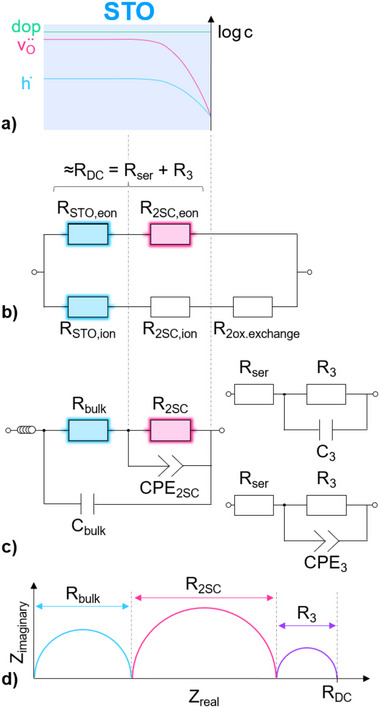
a) Schematic illustration of the depletion of holes and vacancies in the space charge region in STO. b) Equivalent circuit showing only the resistive contributions of the measured impedance data, with mixed ionic and electronic STO bulk (*R*_STO, eon_ and *R*_STO, ion_), ionic and electronic space charge contributions (*R*_2SC, eon_ and *R*_2SC, ion_) and the electrode resistance of the surface oxygen exchange reaction (*R*_2ox.exchange_). c) Equivalent circuit used to fit the high frequency STO and the space charge feature (l.h.s.); with the latter being predominantly caused by electronic species. The third low frequency feature was fitted separately using either a parallel RC or R‐CPE element (r.h.s.). Colors in b and c indicate the same feature in the spectra (blue being the high frequency arc, pink being the medium frequency arc). d) Exemplary illustration of an impedance spectrum, where the quantities shown in c) are marked accordingly.

**Figure** **2** gives a first overview on typical impedance spectra at around 1 · 10^−1^ bar (a) and ca. 2 · 10^−7^ bar (b) measured for LSF, LSM and LSCr on top of STO at 500 °C. At high pressures two arcs are visible, while at low pressures a third arc at low frequencies comes into play. The relative importance of the second arc shrinks toward low *p*(O_2_) and it is barely visible at all for LSF at the *p*(O_2_) of Figure 2b. The high frequency arc, which is of very similar size for all measured samples, is the STO single crystal bulk feature arising from the bulk/mixed ionic and electronic conductivity of STO. Its capacitance results in a relative permittivity of around 150‐160, which is a typical STO value at 500 °C found in literature.^[^ ^45^, ^46^ ^]^ The mid frequency feature was investigated in detail at high *p*(O_2_) in reference^[^ ^42^ ^]^ and can be attributed to the interfacial space charge in STO, induced by the contact with the MIEC layer. It reflects the electronic resistance caused by the depletion of holes in the corresponding space charge layer, cf. **Figure** **3**a. The space charge feature for LSF is much smaller than for LSCr or LSM and even at high *p*(O_2_) it is only a shoulder of the high frequency bulk arc. A more thorough discussion further justifying the assignment of the space charge feature is given below.

The low frequency semicircle is caused by the STO stoichiometry polarization occurring due to the severe blocking of ion transport at the MIEC by slow oxygen surface exchange and/or the interfacial space charges with strong oxygen vacancy depletion, cf. Figure 3a. This ion blocking leads to the situation that the low frequency limit represents the overall resistance of the hole conduction in the nominally undoped, but effectively slightly acceptor doped STO single crystal.^[^ ^18^ ^]^ The same three arcs were observed for STO with Pt electrodes in reference^[^ ^18^ ^]^ and are also discussed in reference^[^ ^47^ ^]^ for polycrystalline STO with space charges at grain boundaries rather than interfacial (electrode) space charge regions. In our study we are particularly interested in the space charge arc (=second arc) and its partial pressure dependence. However, for its interpretation also the size of the first arc and the low frequency limit (DC resistance) are of relevance. The resistive contributions are sketched in Figure 3b with ionic (ion) bulk, space charge and electrode (oxygen exchange) resistances in parallel to the electronic (eon) bulk and space charge resistance. It is also indicated which resistances determine which part of the impedance spectrum. A more detailed argumentation supporting this interpretation is given below.

The high frequency STO arc and the space charge feature were fitted using a nested circuit consisting of a parallel RC and a parallel R‐CPE element, shown in Figure 3c, where *R*_bulk_ is the mixed ionic and electronic resistance of the STO single crystal and *C*_bulk_ is the dielectric bulk capacitance of STO. *R*_2SC_ and *C*_2SC_ are the resistance and capacitance, respectively, of the space charges at both STO|MIEC interfaces. To assure better fit quality, a constant phase element CPE_2SC_ was used instead of a simple capacitor to fit the space charge features. Compared to two serial RC‐elements, the nested circuit takes into account of the fact that the capacitive charge of the interfacial space charge in STO and the charge of the geometrical capacitance of the sample are located in one and the same MIEC layer.^[^ ^48^ ^]^ Further, a nested circuit leads to more consistent space charge capacitance values, especially for LSF. This has been discussed in more detail in a previous work.^[^ ^42^ ^]^ However, for impedance spectra where the space charge feature was no longer clearly noticeable, such as for LSF in Figure 2b, or as later shown for LSM and LSCr at even lower *p*(O_2_), the fitting procedure was performed without the parallel R‐CPE element describing the space charge region.

Since the low frequency semicircle was only used to extract the DC resistance, i.e., the low frequency intercept of the fitting curve with the x‐axis, it was separately quantified by simply using a parallel RC element with a serial resistive offset *R*_ser_, where *R*_3_ and *C*_3_ are the resistance and capacitance of the low frequency semicircle. The sketch in Figure 3d further illustrates the meaning of *R*_bulk_, *R*_2SC_, *R*_3_ and *R*_DC_. Alternatively, this arc was fitted using a parallel R‐CPE element, with CPE_3_ being the constant phase element for the low frequency fit, to estimate the error margin with regard to the determined *p*(O_2_). For some oxygen partial pressures this low frequency feature could have been fitted by simply adding a third RC element in series to the nested circuit. However, especially in the mid *p*(O_2_) range a more complex equivalent circuit, i.e., a transmission line model,^[^ ^18^ ^]^ would often be needed to fit the entire spectrum in a simple run. Since for this study mainly the DC resistance (and *R*_SC_) is of interest, the circuits described above were deemed sufficient to extract the DC resistance of the sample. In case of impedance spectra which did not exhibit the low frequency STO semicircle, the DC resistance was either obtained by the intercept of the x‐axis with the fit of the space charge feature or, if both the space charge resistance feature and the low frequency STO semicircle were non‐existent, the DC resistance was obtained from the intercept of the high frequency STO arc fit with the x‐axis (this is the case for very reducing conditions).

### 3.2 Determination of the Relevant Oxygen Partial Pressure

For the determination of the oxygen partial pressure at the sample, the STO single crystal was used as a *p*(O_2_) sensor. This procedure was possible since very detailed conductivity and defect concentration data for such STO single crystals are available from an earlier study.^[^ ^18^ ^]^ Moreover, the DC resistance almost exclusively reflects the electronic hole conduction across the sample. This is partly due to the fact that the oxygen exchange resistance of vacancy poor MIEC materials is generally very high. For example LSM exhibits oxygen exchange resistances far above our DC resistances.^[^ ^49^, ^50^ ^]^ Moreover, the very severe oxygen vacancy depletion of the doubly charged oxygen vacancies leads to a high ionic space charge resistance, which blocks ions also in case of LSF on STO. By extracting the DC resistance *R*_DC_ and the electronic space charge resistance *R*_2SC_ through fitting the circuit models to the impedance data, the electronic bulk conductivity σ_eon,b_ of STO was calculated as

(1) $$\sigma_{\mathrm{eon},b}=\frac{1}{\rho_{\mathrm{eon},b}}=\frac{1}{\left(R_{\mathrm{DC}}-R_{2\mathrm{SC}}\right)A/d}$$

where ρ_eon,b_ is the electronic bulk resistivity of the STO single crystal, *A* is the area of the sample and *d* is the thickness of the single crystal. The oxygen partial pressure was then calculated by comparing the measured electronic conductivity with the hole (h) and electron (e) conductivities predicted by the defect chemical model of 24 ppm singly charged acceptor doped STO^[^ ^18^ ^]^ at 500 °C, more specifically by considering the regions of Brouwer diagrams where both holes and electrons exhibit a slope of $\frac{1}{4}$ or $-\frac{1}{4}$, respectively.

**Figure 4:**
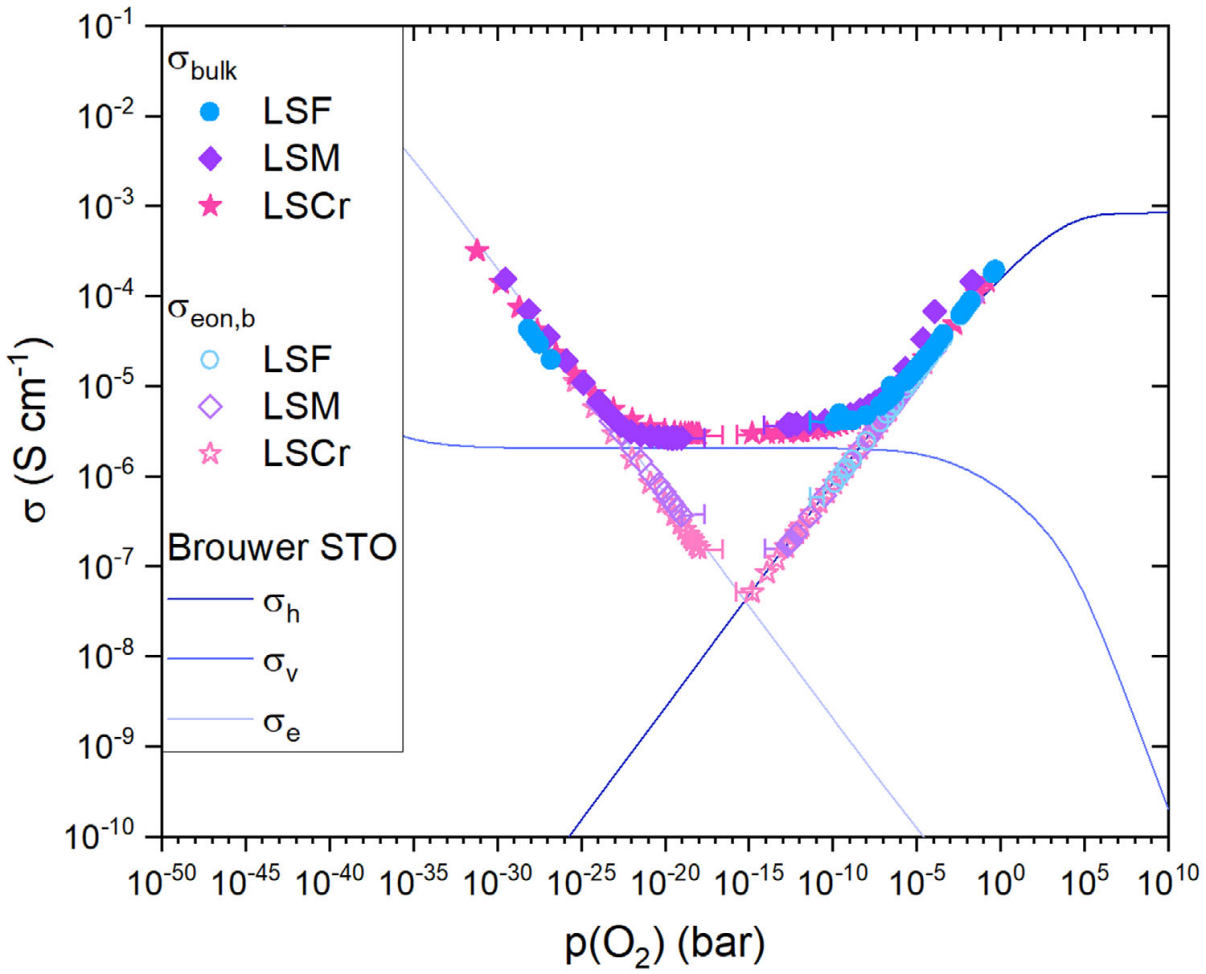
Mixed ionic and electronic conductivity σ_bulk_ and the electronic bulk conductivity σ_eon,b_ of STO plotted against the calculated *p*(O_2_). The diagram also shows conductivities calculated from the defect chemical model in reference,^[^ ^18^ ^]^ i.e., the Brouwer diagram for 24 ppm singly acceptor doped STO at 500 °C.

In order to validate the calculated oxygen partial pressures, the corresponding σ_eon,b_ and $\sigma_{\mathrm{bulk}}=\frac{1}{R_{\mathrm{bulk}}}\frac{d}{A}$ are plotted in the Brouwer‐type diagram for doped STO with 24 ppm singly charged acceptors at 500 °C^[^ ^18^ ^]^ shown in **Figure** **4**. The values of the measured bulk conductivity σ_bulk_ are in very good agreement with the predicted conductivity in all regimes. In the oxidizing partial pressure regime, σ_bulk_ of all investigated samples follows the hole conductivity σ_h_ and exhibits a slope of $\frac{1}{4}$. In the intermediate oxygen partial pressure range, σ_bulk_ stays almost constant for a few orders of magnitude of *p*(O_2_) and agrees very well with the ionic conductivity. This plateau is also the main reason why the determination of the oxygen partial pressure cannot be performed solely through the comparison of σ_bulk_ with the Brouwer diagram. For reducing oxygen partial pressure, σ_bulk_ is in good agreement with the electron conductivity σ_e_ and exhibits a slope of $-\frac{1}{4}$.

The error bars in the mid *p*(O_2_) range arise from difficulties in extracting an accurate value of σ_eon,b_ from impedance data in that region, particularly due to Nyquist plots no longer showing semicircle‐like features. (Exemplary impedance data is shown below.) As a result, data points are not shown in the partial pressure range of ca. 10^−15^ to 10^−20^ bar. The error bars simply reflect the differences arising from the two fit approaches, using a parallel RC element or a parallel R‐CPE element to extract *R*_DC_. Both resulting σ_eon,b_ values where then used to calculate the *p*(O_2_), which correspond to the endpoints of the error bars. In Figure 4 it is also visible that for LSM some slight deviations from ideality are present in the high *p*(O_2_) range, likely due to non‐ideal spectra shapes. This is probably the cause for the nominally lower *p*(O_2_) of LSM in Figure 2a.

### 3.3 Detailed Analysis of Impedance Spectroscopic Measurements

**Figure 5:**
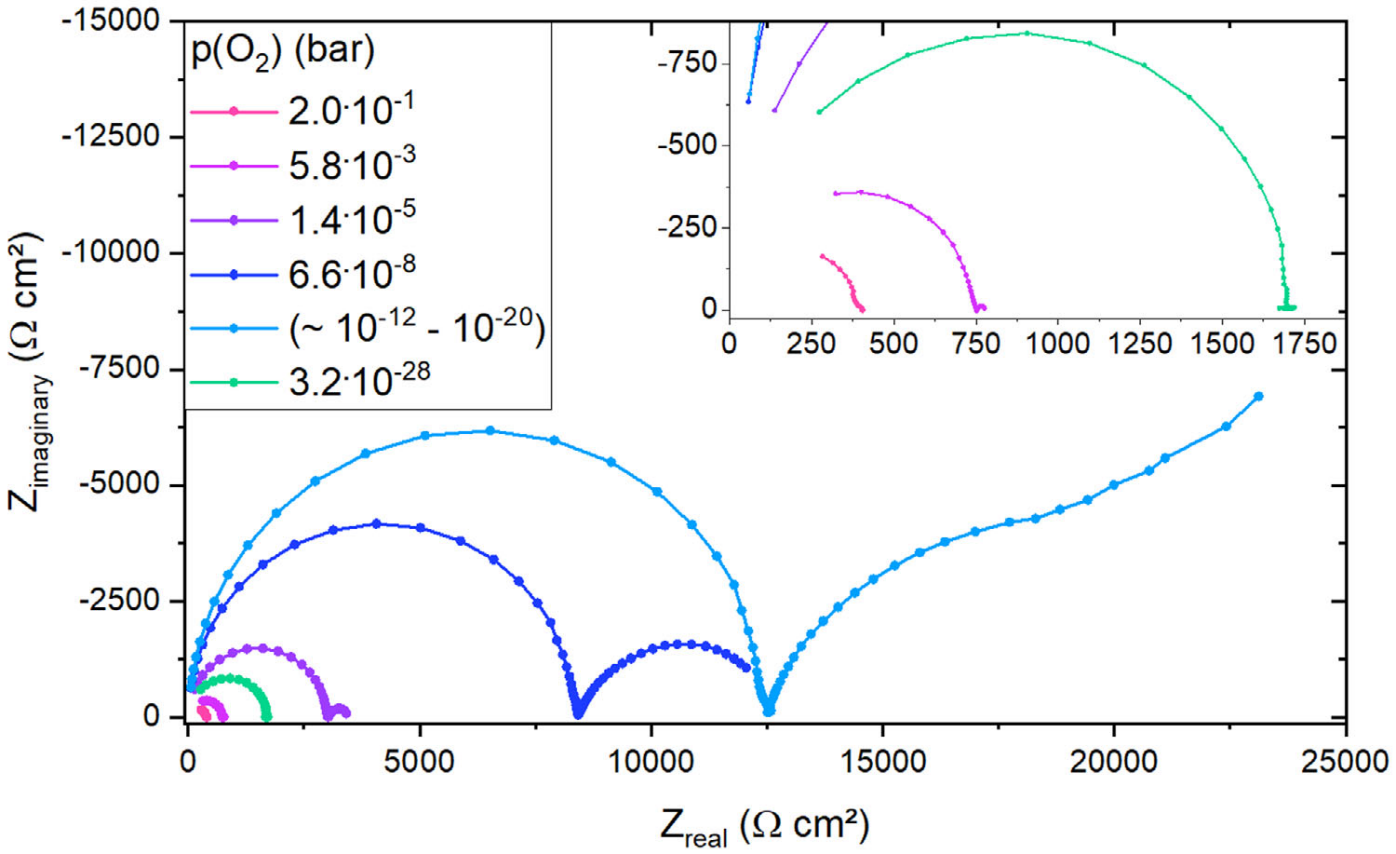
Impedance data for LSF on STO at various oxygen partial pressures at 500 °C. The inset of the figure shows a closer look at impedance data measured for very oxidizing or very reducing partial pressures. The partial pressure for the impedance data labeled as (∼ 10^−12^‐10^−20^) was not clearly determinable.

Measured impedance data of LSF on STO are shown in **Figure** **5** for a broad *p*(O_2_) range. At very oxidizing *p*(O_2_), as shown for 2 · 10^−1^ bar, the impedance data for LSF display parts of the high frequency STO bulk arc and the space charge feature as a small shoulder of this bulk. The STO low frequency semicircle is not present. This is in accordance with the partial conductivities shown in Figure 4: As long as σ_h_ ≫ σ_v_ the blocking of the ionic current does not lead to a visible effect and σ_eon,b_ = σ_bulk_. Toward lower *p*(O_2_), e.g., 1.4 · 10^−5^ bar in Figure 4, the bulk resistance increases and the third semicircle appears since the ionic conductivity approaches the same order of magnitude as the electronic conductivity. Please note that the third arc cannot be caused by the space charge, since the corresponding (chemical) capacitance is by far too large. Its relative importance increases because of the increasing ionic transference number. For these oxygen partial pressures the very small space charge feature strongly overlaps with the STO bulk arc, making analysis of the space charge properties unreliable from approximately 1 · 10^−5^ bar toward lower *p*(O_2_).

At oxygen partial pressures between approximately 10^−12^ to 10^−20^ bar, an accurate determination of *p*(O_2_) was not possible due to problems in extrapolating the impedance curve to achieve the DC resistance. Toward even more reducing conditions, the low frequency feature begins to decrease until it is no longer discernible. This reflects the increase of the electronic transference number, in accordance with the $-\frac{1}{4}$ slope of electrons in the Brouwer diagram. At very reducing conditions, as shown for 3.2 · 10^−28^ bar, impedance data of the LSF sample only exhibits the high frequency STO bulk feature.

**Figure 6:**
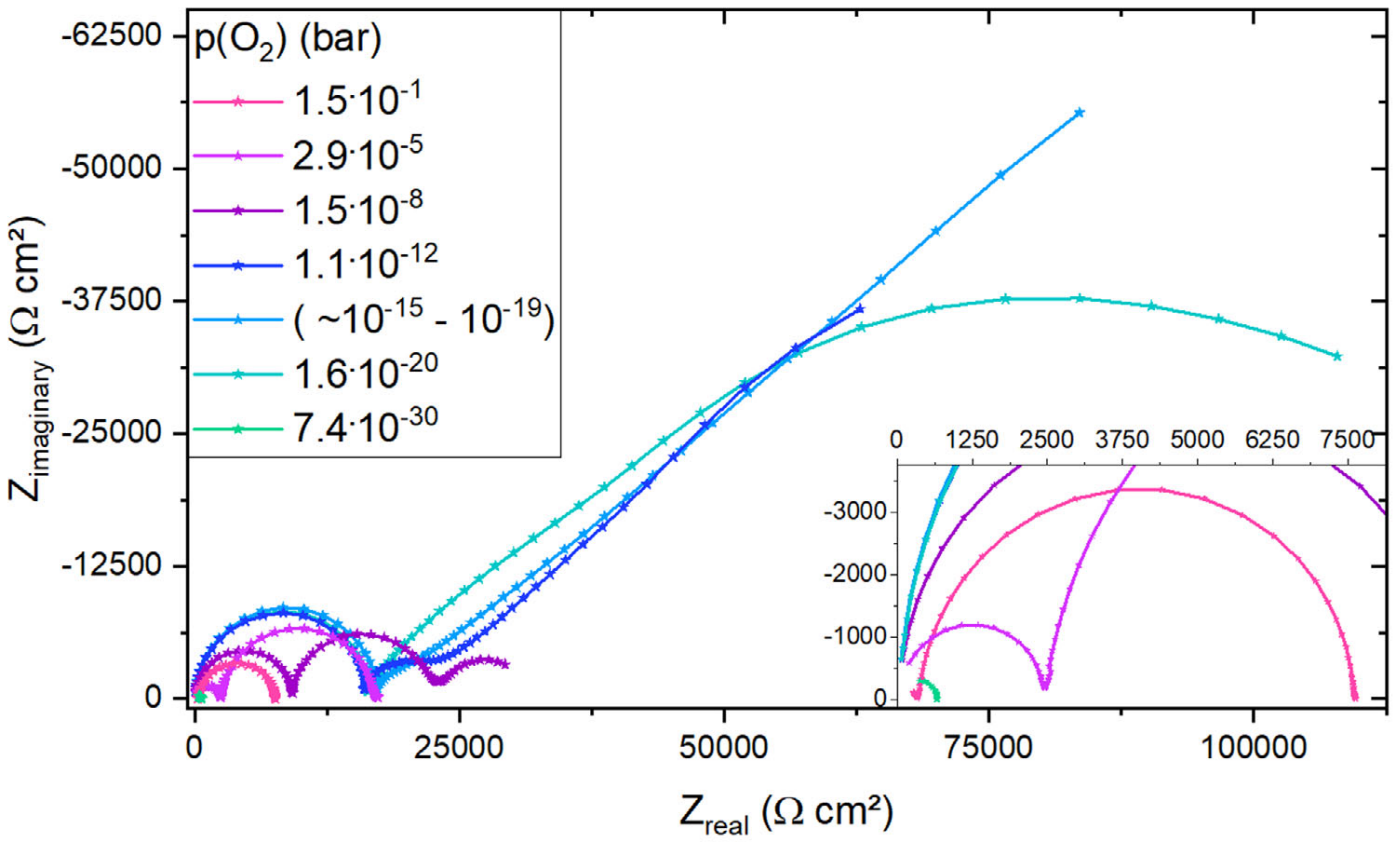
Impedance data for LSCr on STO in a broad oxygen partial pressures at 500 °C. The inset of the figure shows a closer look at impedance data measured for very oxidizing or very reducing partial pressures. The partial pressure for the impedance data labeled as (∼ 10^−15^‐10^−19^) was not clearly determinable.

Impedance data of the LSCr sample is plotted in **Figure** **6**. At high oxygen partial pressures, as shown for 1.5 · 10^−1^ bar, the impedance data of the LSCr sample exhibits only two features: the high frequency bulk STO arc and a very pronounced space charge feature. Toward lower oxygen partial pressures, such as at 2.9 · 10^−5^ bar, both the STO arc and the space charge feature increase in size, however, the STO bulk arc shows a more pronounced increase compared to the space charge feature. In addition, the low frequency STO semicircle becomes detectable. With even lower oxygen partial pressure, the low frequency semicircle increases significantly in size, whereas the space charge feature starts to decrease from roughly 10^−7^ bar toward lower *p*(O_2_). This leads to the situation that the small space charge feature merges with the still increasing low frequency semicircle, see 1.1 · 10^−12^ bar, where the space charge feature is still present as a small shoulder of the low frequency feature, and 10^−15^‐10^−19^ bar, where the space charge feature is no longer separable from the low frequency semicircle. As a consequence, the space charge properties of the LSCr sample can only be analyzed down to approximately 10^−13^ bar. Further, in the range of approximately 10^−15^ bar to 10^−19^ bar, a *p*(O_2_) determination is not possible, as the low frequency extrapolation of the impedance data fails. Similar to the LSF sample, both the high frequency and the low frequency arc decrease in size toward very reducing conditions. At very low *p*(O_2_), as shown for 7.4 · 10^−30^ bar, impedance spectra display only the high frequency STO bulk arc.

**Figure 7:**
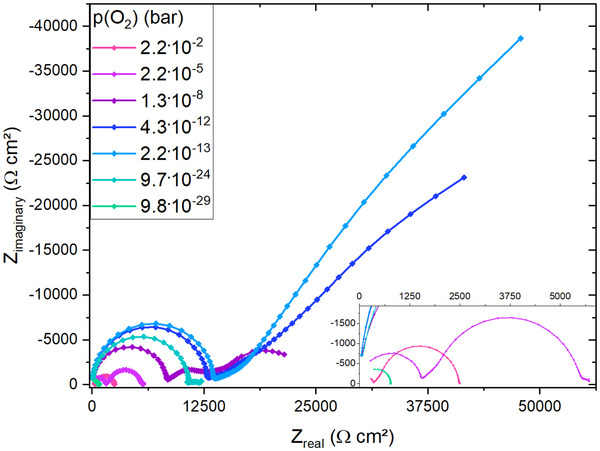
Impedance data for LSM on STO in a broad oxygen partial pressures at 500 °C. The inset of the figure shows a closer look at impedance data measured for very oxidizing or very reducing partial pressures.

Impedance data for LSM are given in **Figure** **7**. Overall the trends of the impedance data of LSM are similar to the LSCr sample, even though the space charge arc is less ideal toward high frequencies. Exact reasons behind this non‐ideality remained unknown. Since this does not have a very pronounced effect on the determination of *R*_2SC_, it was not further considered. The oxygen partial pressure dependence of all arcs of the LSM sample is very similar to the LSCr sample. At very high *p*(O_2_), only the high frequency STO and space charge feature are present, as shown for 2.2 · 10^−2^ bar. Toward lower partial pressures, both features increase in size and the low frequency semicircle becomes visible. The overlap between the space charge feature and the low frequency arc occurs also for LSM in the intermediate *p*(O_2_) range, see 1.3 · 10^−8^ bar and 4.3 · 10^−12^ bar. Consequently, the space charge arc of LSM|STO can only be analyzed down to roughly 10^−11^ bar. Similar to the LSF and LSCr sample, both the high frequency and the low frequency arcs decrease in size toward very reducing conditions and at very low *p*(O_2_), as shown for 9.8 · 10^−29^ bar, only the high frequency STO bulk arc is present.

### 3.4 Analysis of the Resistances and Capacitances

**Figure 8:**
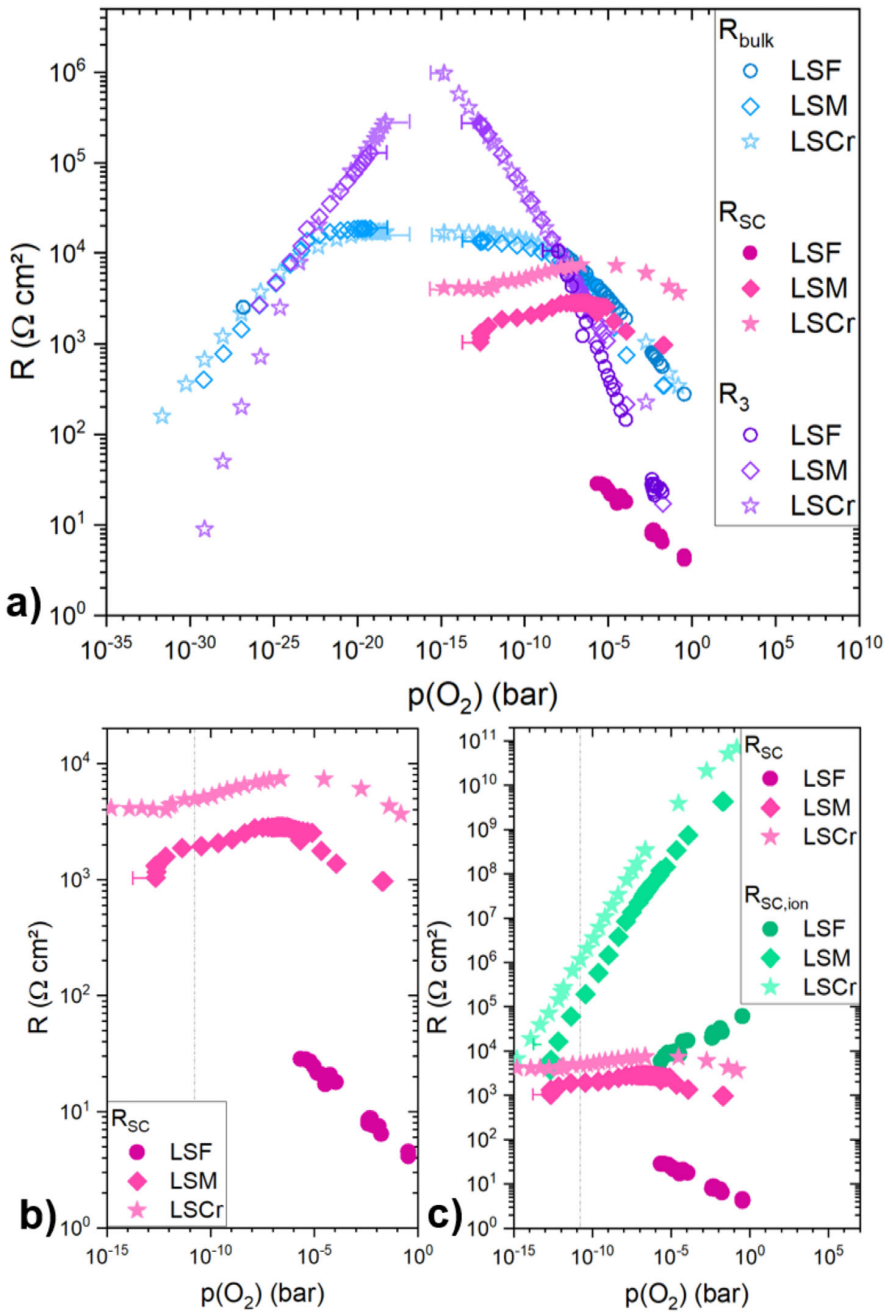
a) The resistance of the STO bulk *R*_bulk_, the resistance of one space charge zone *R*_SC_ and the resistance attributed to the stoichiometry change in STO *R*_3_ of the investigated samples with MIECs (LSF, LSM and LSCr) at 500 °C plotted against the *p*(O_2_). b) Close‐up of *R*_SC_ only. c) Comparison of the measured (electronic) resistance of the space charge zone *R*_SC_ and the estimated ionic resistance of the space charge zone *R*_SC, ion_ (Equation (5)). The gray dashed line in b) and c) indicates the lowest *p*(O_2_) were the space charge arc is separable from the low frequency semicircle.

The fitted resistances of all investigated samples are given in **Figure** **8**. For the entire *p*(O_2_) range of all measured samples *R*_bulk_ initially increases with decreasing *p*(O_2_), plateaus in the intermediate *p*(O_2_) range and then again decreases, all in accordance with the already plotted total mixed ionic and electronic conductivity of the STO single crystal, shown in Figure 4. $R_{\mathrm{SC}}\left(=\frac{1}{2}R_{2\mathrm{SC}}\right)$, see Figure 8b, is the resistance of one interfacial space charge and reflects the hole depletion in STO close to the MIEC interface. The space charge resistance is only visible in the *p*(O_2_) range where hole conduction dominates the electronic conductivity but covers also ranges where the bulk conduction itself is primarily ionic. Very prominent is the difference of the space charge resistances between the different samples. While LSF exhibits rather small values with the smallest measured space charge resistance being 4.5Ωcm^2^ at 2.5 · 10^−1^ bar, *R*_SC_ of LSM and LSCr are rather high, e.g. 964.1Ωcm^2^ and 3662.1Ωcm^2^ at 2.2 · 10^−2^ bar and 1.5 · 10^−1^ bar, respectively. At high oxygen partial pressures, the space charge resistances substantially increase with decreasing *p*(O_2_). At lower *p*(O_2_) (only accessible for LSM and LSCr), on the other hand only a rather slight decrease is visible (ca. a factor 2 over almost 10 orders of magnitude in *p*(O_2_) change). Figure 8c shows a comparison between the measured electronic space charge resistance *R*_SC_ and the calculated ionic space charge resistance *R*_SC, ion_, with the latter being estimated from the space charge potential deduced from the electronic contributions, see below. At high oxygen partial pressures, *R*_SC, ion_ is by several orders of magnitude larger than the measured *R*_SC_ and thus the space charge zone is far more blocking for ionic than for electronic species. Toward intermediate *p*(O_2_) *R*_SC, ion_ decreases, but even for the lowest partial pressure where the space charge feature can be analyzed, *R*_SC, ion_ is still an order of magnitude larger than *R*_SC_.

*R*_3_, being the resistance of the low frequency semicircle, is overall very similar for all investigated samples. This is expected as we attribute this feature to the blocking of ions in the nominally identical STO single crystals. More specific

(2) $$\begin{array}{c} R_{3}=R_{\mathrm{DC}}-R_{\mathrm{ser}}\approx R_{\mathrm{DC}}-R_{\mathrm{bulk}}-R_{2\mathrm{SC}}=\\ R_{\mathrm{eon},b}-R_{\mathrm{bulk}}=R_{\mathrm{eon},b}-\frac{R_{\mathrm{eon},b}\cdot R_{\mathrm{ion},b}}{R_{\mathrm{eon},b}+R_{\mathrm{ion},b}} \end{array}$$

In the primarily ion conducting regime of the STO bulk the last term in Equation (2) vanishes and *R*_3_ is close to *R*_eon,b_ and peaks at the *p*(O_2_) where hole conduction changes to electron conduction. For the LSCr sample this is around 10^6^ Ωcm^2^ at 1.6 · 10^−15^ bar. For predominant hole or electron conduction in the bulk of STO the last term approaches *R*_eon,b_ and thus *R*_3_ becomes small and finally non‐measurable.

**Figure 9:**
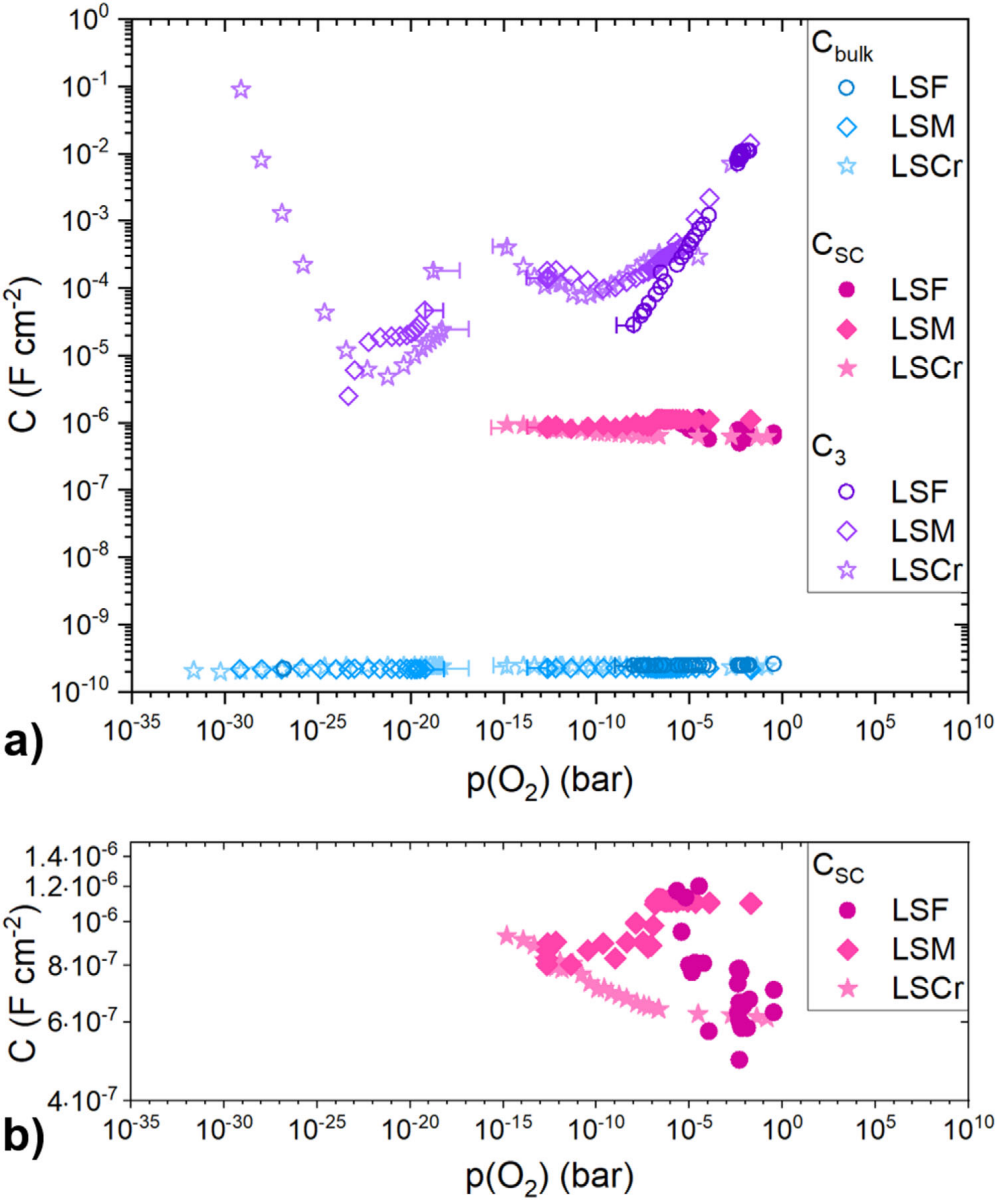
a) The capacitance of the STO bulk *C*_bulk_, the capacitance of one space charge zone *C*_SC_ and the capacitance *C*_3_ caused by the stoichiometry change in STO and related to the chemical capacitance of STO for all investigated MIECs (LSF, LSM, and LSCr) at 500 °C plotted against the *p*(O_2_). b) Close‐up of *C*_SC_.

The capacitances extracted from impedance measurements are plotted against the investigated oxygen partial pressure range in **Figure** **9**. *C*_bulk_ yields ca. 2.3 · 10^−10^ F/cm^2^ for all samples. As mentioned previously, this corresponds to a relative permittivity of 150‐160, which is typical for STO at 500 °C.^[^ ^45^, ^46^ ^]^ *C*_SC_ is the capacitance of one interfacial space charge calculated from the fit parameter *C*_2SC_ by multiplying by 2. Since the space charge features were fitted using a constant phase element with an impedance of $Z_{\mathrm{CPE}}=\frac{1}{{\left(j\omega \right)}^{\alpha }Q}$, *C*_2SC_ was calculated according to $C={\left(R^{1-\alpha }\cdot Q\right)}^{\frac{1}{\alpha }}$.^[^ ^51^ ^]^ For all samples *C*_SC_ is around 1 · 10^−6^ F/cm^2^ and remains rather constant throughout the investigated *p*(O_2_) range. Using the bulk permittivity of STO, this capacitance value corresponds to a thickness of 130 to 140 nm. This strongly supports the assignment to a space charge, since these values are close to the values expected for interfacial space charge regions in STO with the given doping concentration, cf. discussion in reference^[^ ^18^, ^35^, ^42^ ^]^ and below. More specific, the capacitance *C*_SC_ for LSCr is 6.3 · 10^−7^ F/cm^2^ at 1.5 · 10^−1^ bar and increases slightly to 9.3 · 10^−7^ F/cm^2^ at 1.6 · 10^−15^ bar. *C*_SC_ for LSF yields 6.5 · 10^−7^ F/cm^2^ at 2.0 · 10^−1^ bar and increases to 1 · 10^−6^ F/cm^2^ at 2.2 · 10^−6^ bar. Both cases suggest a slight decrease of the space charge thickness with decreasing *p*(O_2_), which might be caused by a decrease of the space charge potential, see below. Only for LSM a slight decrease of *C*_SC_ is found from 1.1 · 10^−6^ F cm^2^ at 2.2 · 10^−2^ bar to 8.0 · 10^−7^ F/cm^2^ at 2.2 · 10^−13^ bar. However, this decrease for LSM might also be due to fit inaccuracies that arise from separating the small space charge feature and the strongly distorted third arc.

As already discussed for the similar cases of Pt electrodes^[^ ^18^ ^]^ and polycrystalline STO,^[^ ^47^ ^]^ *C*_3_ is related to the stoichiometry change in STO due to ion blocking and thus represents a chemical capacitance. This capacitance shows by far the largest *p*(O_2_) dependence. At 1 · 10^−3^ bar it yields 1 · 10^−2^ F/cm^2^ and decreases with decreasing *p*(O_2_) until approximately 10^−10^ bar. Deviations between LSF and LSM or LSCr below 10^−5^ bar might be fit artifacts. At intermediate partial pressures, *C*_3_ increases slightly and seems to exhibit a peak around 10^−16^ bar. At very low pressures *C*_3_ of the LSCr sample strongly increases again. This curve shape was also found for the STO single crystals with Pt electrodes in reference^[^ ^18^ ^]^ and there a detailed discussion and interpretation is given. In short, the chemical capacitance reflects the concentration of the most important minority charge carrier and thus decreases with the hole concentration at high *p*(O_2_) and increases with the electron concentration at low *p*(O_2_). The small peak or flattening of the capacitance in the intermediate *p*(O_2_) range suggests the presence of additional mid gap electronic states.^[^ ^18^ ^]^

### 3.5 The Space Charge Potential

Assuming validity of the drift‐diffusion model and a Schottky‐approximation, the space charge potential Δϕ in STO can be calculated from the area‐specific electronic bulk resistance *R*_eon,b_ and the area‐specific electronic resistance of one space charge *R*_SC_ of STO in accordance with

(3) $$\frac{R_{\mathrm{SC}}\frac{1}{w_{\mathrm{SC}}}}{R_{\mathrm{eon},b}\frac{1}{d}}=\frac{\exp\frac{e\Delta \phi }{kT}}{\frac{2e\Delta \phi }{kT}}$$

where e is the elementary charge, k is the Boltzmann constant and T is the temperature.^[^ ^42^, ^52^ ^]^ As already discussed above, *R*_eon,b_ is determined via *R*_eon,b_ = *R*_DC_ − *R*_2SC_. The Schottky approximation defines the thickness of the space charge w_SC_ as

(4) $$w_{\mathrm{SC}}=\sqrt{\frac{2\varepsilon_{r}\varepsilon_{0}\left|\Delta \phi \right|}{eN_{d}}}$$

where ε_0_ is the relative permittivity, ε_*r*_ is the relative permittivity of STO and *N*_d_ is the dopant concentration. Equation (4) also implies that the space charge zones in the highly Sr‐doped MIECs are very thin (<1 nm) and thus electrostatic potential changes therein are essentially negligible. As a results the space charge in STO has to be almost identical to the electrostatic potential difference between the MIEC and the STO bulk, i.e. Δϕ ≈ ϕ^MIEC^ − ϕ^STO^, when disregarding any interfacial dipole layer or additional charge trapping at the core of the interface.

**Figure 10:**
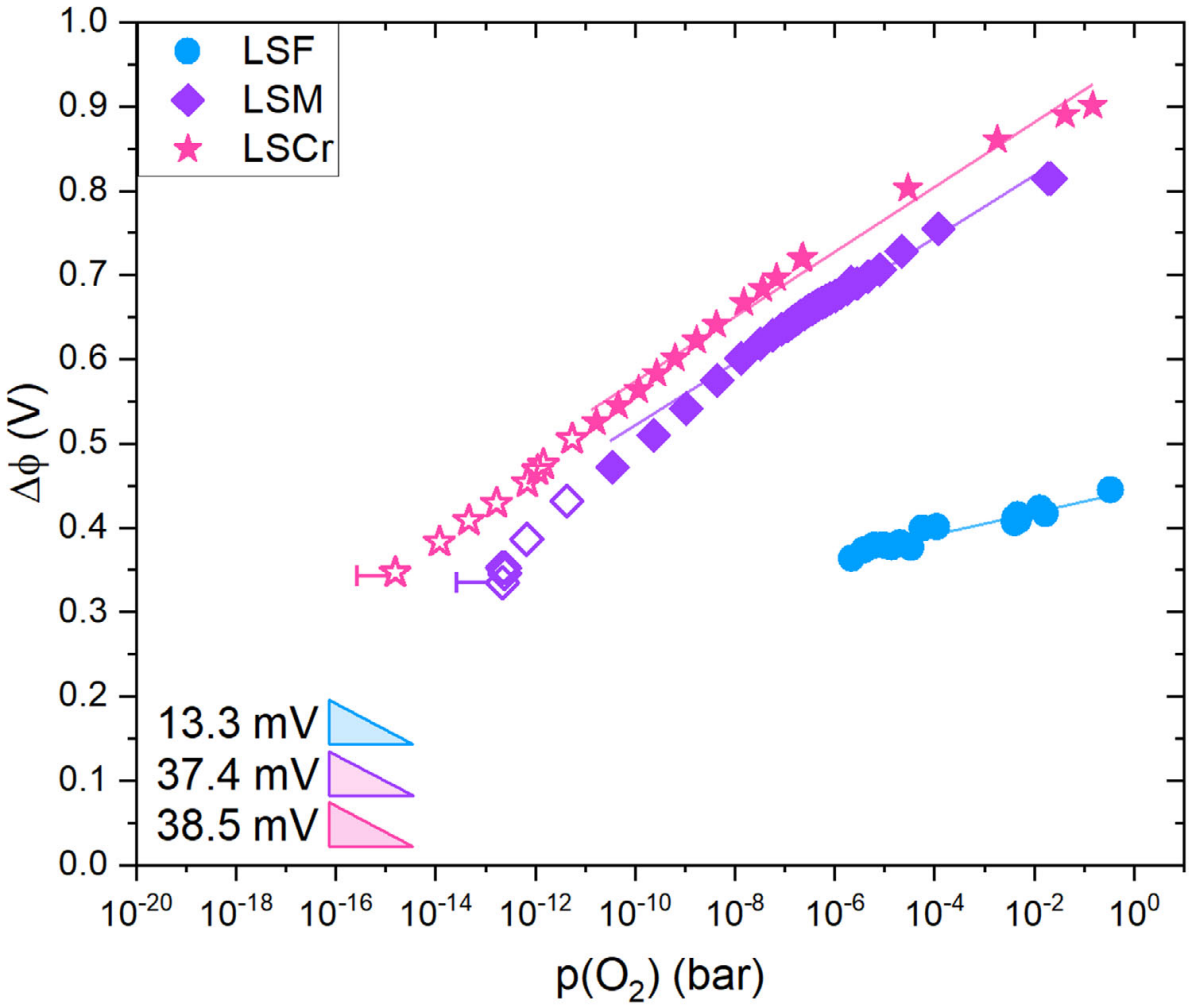
Δϕ in dependence of the oxygen partial pressure. The closed symbols represent the data used for determining the slopes. The slope is given per decade *p*(O_2_) for the partial pressure range indicated by the closed symbols.

For Δϕ calculations, ε_*r*_ was assumed constant throughout the entire STO single crystal, including the space charge regions. For nominally identical STO single crystals a dopant concentration of 24 ppm singly charged acceptor dopants was found,^[^ ^18^ ^]^ which was therefore used as N_*d*_ value in Equation (4) to determine w_SC_. Δϕ is then obtained by solving Equation (3) numerically. The calculated space charge potentials for the interfaces between STO and LSF, LSM and LSCr are presented in **Figure** **10**. The space charge potential for LSF is 445 mV at 2 · 10^−1^ bar. Δϕ for LSM is larger with 814 mV at 2.2 · 10^−2^ bar. The largest Δϕ was found for LSCr with 901 mV at 1.5 · 10^−1^ bar. These Δϕ values and the Δϕ trend LSF < LSM < LSCr are in agreement with previous space charge investigations.^[^ ^42^ ^]^ With decreasing *p*(O_2_), Δϕ decreases by 37.4 mV per decade *p*(O_2_) for LSM and by 38.5 mV for LSCr. The slope of Δϕ for LSM slightly steepens toward intermediate *p*(O_2_), but this might be due to the space charge feature being increasingly overlapped by the low frequency feature in the impedance data from ca. 10^−10^ bar to lower partial pressures. Hence, only data points down to 10^−10^ bar were used for determining the slopes of Δϕ.

This also means that the rather non‐trivial shapes of the resistance vs. *p*(O_2_) curves in Figure 8 finally translate into a simple *p*(O_2_) dependence of Δϕ. The model considerations in the next chapter show that a simple defect model of STO and the MIEC suggests a slope of 38.3 mV for LSM and LSCr and thus almost perfectly describe the measured dependencies. For Δϕ of the LSF sample, only a much smaller *p*(O_2_) regime can be considered and the resulting slope is also much smaller (13.3 mV per decade *p*(O_2_)). At least partly the smaller slope can be attributed to the defect model with a varying hole concentration in LSF in the respective *p*(O_2_) range as detailed below. However, also some fitting difficulties of the small space charge feature might play a role.

The ionic space charge resistance is experimentally not accessible in our study. However, it can be estimated from Δϕ through

(5) $$\frac{R_{\mathrm{SC},\mathrm{ion}}\frac{1}{w_{\mathrm{SC}}}}{R_{\mathrm{STO},\mathrm{ion}}\frac{1}{d}}=\frac{\exp\frac{2e\Delta \phi }{kT}}{\frac{4e\Delta \phi }{kT}}$$

where *R*_STO, ion_ is the ionic bulk resistance of STO, which is constant in a broad *p*(O_2_) range and can be approximated by the measured average *R*_bulk_ found in the intermediate *p*(O_2_) range. *R*_SC, ion_ values are compared to the experimental *R*_SC_ in Figure 8c. At intermediate *p*(O_2_), *R*_SC, ion_ is by one order of magnitude larger than *R*_SC_ and even several orders of magnitude larger at higher *p*(O_2_), meaning that a strong effect of *R*_SC, ion_ on the measured *R*_SC_ is not expected in the given partial pressure range. Hence, *R*_SC_ ≈ *R*_SC, eon_ and *R*_STO, ion_ + *R*_2SC, ion_ ≫ *R*_STO, eon_ + *R*_2SC, eon_ in the entire relevant *p*(O_2_) range.

### 3.6 Model Considerations

The following considerations are based on an extended model introduced in reference.^[^ ^42^ ^]^ The full model, based on measurements in the high *p*(O_2_) range, explains how ionic and electronic charge carriers interact in defining Δϕ and how Δϕ is related to the reducibility of the MIECs. Here, we first summarize the key features of the model needed to explain the *p*(O_2_) dependence of Δϕ.

The normalized oxygen partial pressure *p*(O_2_) (normalized to 1 bar) is related to the atmospheric oxygen chemical potential $\mu_{O_{2}}^{\mathrm{at}}$ through

(6) $$\mu_{O_{2}}^{\mathrm{at}}=\mu_{O_{2}}^{0}+kT\;\ln p\left(O_{2}\right)$$

where $\mu_{O_{2}}^{0}$ is the standard chemical potential. Given sufficiently fast oxygen exchange kinetics, the oxygen chemical potential of a sample $\mu_{O_{2}}^{\mathrm{sample}}$ that is brought into a given atmosphere starts to align with $\mu_{O_{2}}^{\mathrm{at}}$ until equilibrium is reached. For a sample consisting of multiple phases, such as STO|MIEC, each single phase thus equilibrates with the surrounding atmosphere until $\mu_{O_{2}}^{\mathrm{at}}=\mu_{O_{2}}^{\mathrm{STO}}=\mu_{O_{2}}^{\mathrm{MIEC}}$.

The oxygen exchange reaction $\frac{1}{2}O_{2}+v_{O}^{..}⇌2h^{.}$ links the oxygen chemical potential and the chemical potential of point defects, i.e., of the holes and the vacancies, µ_h_ and µ_v_, respectively, through

(7) $$\frac{1}{2}\mu_{O_{2}}+\mu_{v}=2\mu_{h}$$

For both phases being equilibrated with the gas phase, we thus find

(8) $$2\Delta \mu_{h}=\Delta \mu_{v}$$

Please note that Δµ_h_ refers to the difference in the chemical potentials of the holes between STO and the MIEC $\left(\mu_{h}^{\mathrm{MIEC}}-\mu_{h}^{\mathrm{STO}}\right)$. The same meaning applies for the difference in the chemical potential of the vacancies, where $\Delta \mu_{v}=\mu_{v}^{\mathrm{MIEC}}-\mu_{v}^{\mathrm{STO}}$.

If STO and the MIEC are in contact, also a defect chemical equilibrium is finally reached. This, however, requires the formation of a space charge zone. Since this is achieved through the transfer of charged species, an electrostatic potential difference is induced. Thus the equilibrium of charged species, i.e. holes and vacancies, has to be described by the electrochemical potential $\tilde{\mu }=\mu +ze\phi$, with *z* being the charge number. The equilibrium for holes requires ${\tilde{\mu }}_{h}^{\mathrm{STO}}={\tilde{\mu }}_{h}^{\mathrm{MIEC}}$ and thus

(9) $$\Delta \mu_{h}=-e\Delta \phi$$

(Please note: Symbol Δϕ here means ϕ^MIEC^ − ϕ^STO^, but for negligible interfacial charges in space charges in the MIEC it equals the space charge potential in STO, deduced by Equation 3). Our previous work^[^ ^42^ ^]^ discusses in detail that the space charge potential can be described either through ionic or electronic charge carriers, as both the ionic and electronic charge carries, i.e. µ_v_ and µ_h_ in Equation (8) imply the same equilibrium potential difference between the two bulk phases according to

(10) $$-e\Delta \phi =\Delta \mu_{h}=\frac{1}{2}\Delta \mu_{v}$$

Here, we focus on µ_h_ in order to discuss the space charge potential in the hole conducting STO. However, all the considerations can also be made from the viewpoint of oxygen vacancies.

In the most general case the chemical potential can be described by µ = µ^0^ + *kT*ln *a*, with *a* being the activity. In the following, we neglect energetic charge carrier interaction. Without site restriction we thus have *a* = c = defect concentration normalized to the corresponding site concentration. Including site restriction we get $a=\frac{c}{1-c}$. Considering site restrictions for the holes of highly doped MIECs and a dilute situation for the nominally undoped STO, Equation (9) can thus be further detailed as

(11) $$-e\Delta \phi =\Delta \mu_{h}^{0}+kT\ln\frac{c_{h}^{\mathrm{MIEC}}}{c_{h}^{\mathrm{STO}}\left(1-c_{h}^{\mathrm{MIEC}}\right)}$$

The standard chemical potential $\mu_{h}^{0}$ can be used to predict general trends of the space charge potential of different MIECs, e.g. the much larger Δϕ for LSCr compared to LSF.^[^ ^42^ ^]^ However, it is independent of *p*(O_2_) and thus the dependence of Δϕ on the oxygen partial pressure has to be given by the concentration term $\ln\frac{c_{h}^{\mathrm{MIEC}}}{c_{h}^{\mathrm{STO}}\left(1-c_{h}^{\mathrm{MIEC}}\right)}$. The hole concentrations of both STO and the MIEC in general depend on *p*(O_2_) and this is quantified by defect chemical models, i.e., Brouwer diagrams. In order to predict the change in Δϕ in dependence of the partial pressure ($e\frac{d\Delta \phi }{d\ln p\left(O_{2}\right)}$), we thus only need to consider the Brouwer diagrams of both STO and the MIEC.

Δϕ (Equation (11)) can be differentiated with respect to ln *p*(O_2_), which leads to

(12) $$\begin{array}{c} e\frac{d\Delta \phi }{d\ln p\left(O_{2}\right)}=-\frac{d\Delta \mu_{h}^{0}}{d\ln p\left(O_{2}\right)}-\\ kT\left(\frac{d\ln c_{h}^{\mathrm{MIEC}}}{d\ln p\left(O_{2}\right)}-\frac{d\ln c_{h}^{\mathrm{STO}}}{d\ln p\left(O_{2}\right)}-\frac{d\ln\left(1-c_{h}^{\mathrm{MIEC}}\right)}{d\ln p\left(O_{2}\right)}\right) \end{array}$$

with $\frac{d\Delta \mu_{h}^{0}}{d\ln p\left(O_{2}\right)}$ being 0.

For a dilute situation in both the STO and the MIEC this simplifies to

(13) $$e\frac{d\Delta \phi }{d\ln p\left(O_{2}\right)}=-kT\left(\frac{d\ln c_{h}^{\mathrm{MIEC}}}{d\ln p\left(O_{2}\right)}-\frac{d\ln c_{h}^{\mathrm{STO}}}{d\ln p\left(O_{2}\right)}\right)$$

For the following considerations, we divide the Brouwer diagrams into regions, where the slope α of ln *c*_h_ vs. ln *p*(O_2_) is constant and can be defined by a power law ln *c*_h_ = β + αln *p*(O_2_). Thus the slope α is given by $\alpha =\frac{d\ln c_{h}}{d\ln p\left(O_{2}\right)}$. Comparing this with Equation (13) demonstrates, that in dilute cases the oxygen partial pressure dependence of Δϕ can be described by only taking the slopes of the Brouwer diagrams of both STO and the MIEC into consideration. Based on this assumption, three different simplified scenarios may occur: In the simplest case, *c*_h_ stays constant for both materials ($\frac{d\ln c_{h}^{\mathrm{STO}}}{d\ln p\left(O_{2}\right)}=0$ and $\frac{d\ln c_{h}^{\mathrm{MIEC}}}{d\ln p\left(O_{2}\right)}=0$). As a result, $e\frac{d\Delta \phi }{d\ln p\left(O_{2}\right)}$ is 0 and Δϕ does not change with the *p*(O_2_). Secondly, *c*_h_ stays constant for only one of the materials, such as for the MIEC ($\frac{d\ln c_{h}^{\mathrm{MIEC}}}{d\ln p\left(O_{2}\right)}=0$). In this case the *p*(O_2_) dependence is given by $\frac{d\ln c_{h}^{\mathrm{STO}}}{d\ln p\left(O_{2}\right)}$. Assuming the slope for STO is $\frac{1}{4}$, $\frac{e\Delta \phi }{kT}$ vs. ln *p*(O_2_) is also expected to exhibit a slope of $\frac{1}{4}$. At 500 °C this corresponds to a space charge slope of 38.3 mV per decade in *p*(O_2_). Lastly, if the slopes for STO and the MIEC have different values, $\frac{d\ln c_{h}^{\mathrm{STO}}}{d\ln p\left(O_{2}\right)}\neq \frac{d\ln c_{h}^{\mathrm{MIEC}}}{d\ln p\left(O_{2}\right)}$, then Δϕ in dependence of the partial pressure is expected to reflect the difference between the slopes. For a non‐dilute case in the MIEC the situation becomes slightly more complex for varying $c_{h}^{\mathrm{MIEC}}$ due to the final term in Equation (12).

**Figure 11:**
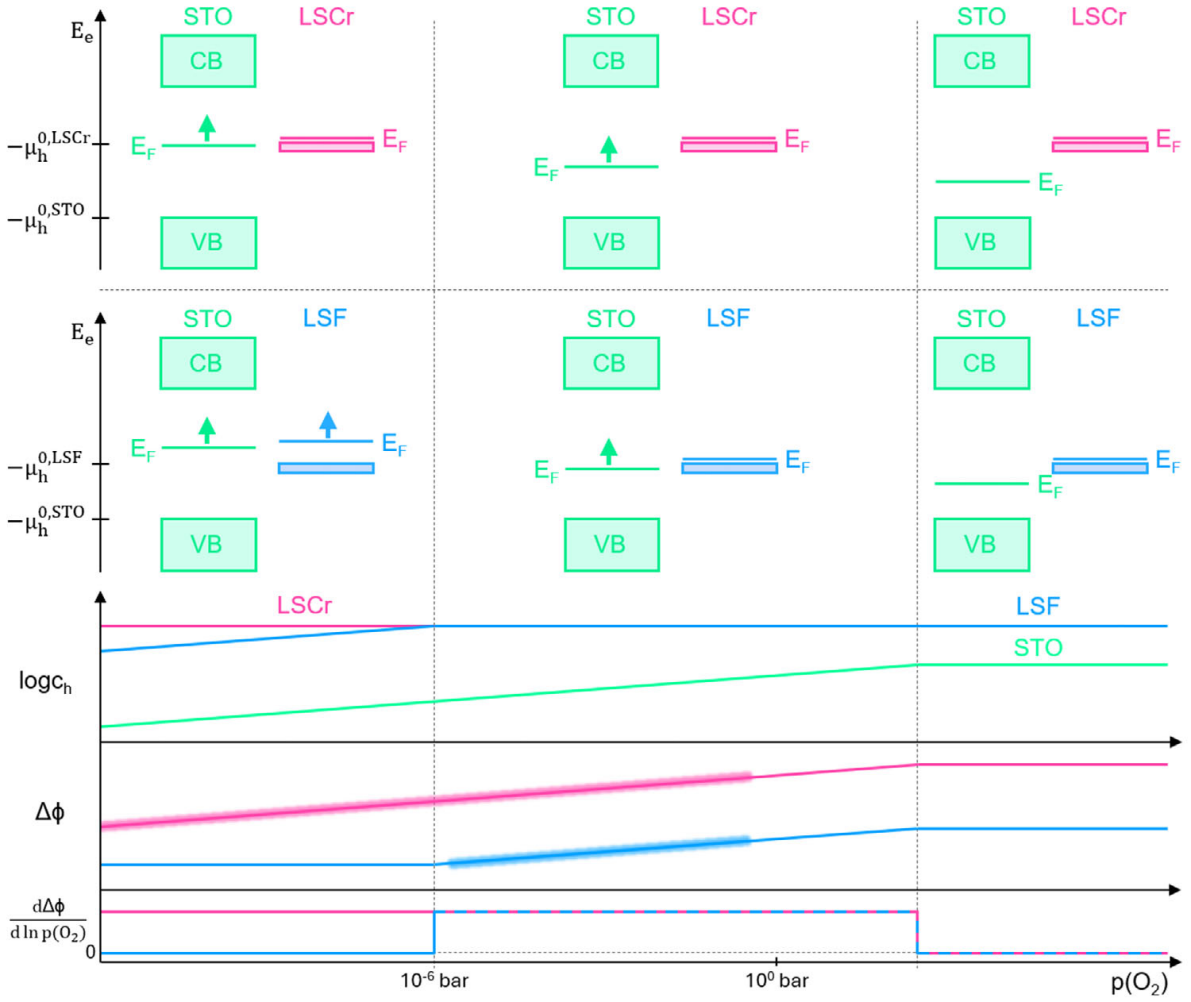
Schematic illustration of the Δϕ dependence on the *p*(O_2_), which is correlated to the change in *c*_h_ of the defect chemical model for both the STO and the MIEC; here exemplarily plotted for LSF and LSCr at the bottom. The Δϕ regions plotted in bold mark the *p*(O_2_) range for LSF or LSCr, where the space charge potential was experimentally accessible. The corresponding band schemes for STO|LSCr (top) and STO|LSF (center) before space charge formation are also given, including the *p*(O_2_) dependent Fermi level E_F_ = −µ_h_. The upward arrows are plotted from the perspective of decreasing *p*(O_2_). $\mu_{h}^{0}$ equals the valence band edge for STO and represents the hole polaron levels for LSF and LSCr, respectively. The width of these polaronic levels is arbitrarily chosen.

We may now apply these considerations to our specific materials in the *p*(O_2_) range where Δϕ is accessible. According to the defect chemical models of STO,^[^ ^18^ ^]^ $c_{h}^{\mathrm{STO}}$ exhibits a slope of $\frac{1}{4}$ throughout the entire investigated *p*(O_2_) range at 500 °C. Defect chemical models for LSF^[^ ^21^, ^53^ ^]^ suggest that at 500 °C $c_{h}^{\mathrm{LSF}}$ stays constant at the dopant concentration from 1 bar to ca. 10^−4^ bar, then bends and reaches a slope of $\frac{1}{4}$ from ca. 10^−9^ bar toward lower *p*(O_2_). Literature data for LSM^[^ ^54^ ^]^ and LSCr^[^ ^55^ ^]^ show that the reducibility of both materials is rather low. $c_{h}^{\mathrm{LSM}}$ and $c_{h}^{\mathrm{LSCr}}$ thus stay constant from 1 bar to 10^−22^ bar and 1 bar to 10^−31^ bar, respectively, and decrease toward even lower partial pressures with a slope of $\frac{1}{4}$. The space charge potential could be measured for LSF between 1 bar and 10^−6^ bar and for LSM and LSCr down to approximately 10^−11^ bar. Hence, we can assume that *c*_h_ for LSM and LSCr is constant ($\frac{d\ln c_{h}^{\mathrm{MIEC}}}{d\ln p\left(O_{2}\right)}=0$) and that the dependence of Δϕ on the oxygen partial pressure is entirely governed by the change of *c*_h_ in STO. This is schematically sketched in **Figure** **11** for LSCr. To understand the Δϕ change for LSF, a simplified defect chemical model was assumed for LSF and is also shown in Figure 11.

The *c*_h_ change with *p*(O_2_) can be also expressed in terms of a band scheme, with Fermi level E_F_ = −µ_h_, see Figure 11. The valence band edge $E_{\mathrm{VB}}^{\mathrm{STO}}$ in STO is $-\mu_{h}^{0,\mathrm{STO}}$, while $-\mu_{h}^{0,\mathrm{MIEC}}$ represents the hole polaron level in the contacting MIEC, e.g., LSCr, assuming polaronic transport rather than band conduction in the highly doped MIECs. The bandgap in STO is 2.8 eV at 500 °C^[^ ^19^ ^]^ and E_F_ lies ca. 0.83 eV above $E_{\mathrm{VB}}^{\mathrm{STO}}$ at 1 · 10^−3^ bar and 500 °C.^[^ ^42^ ^]^ The positions of E_F_ for LSF and LSCr compared to STO (before contacting) reflect essentially the measured Δϕ values, according to Equation (9). The position of the polaron level $-\mu_{h}^{0,\mathrm{LSF}}$ relative to $-\mu_{h}^{0,\mathrm{STO}}=E_{\mathrm{VB}}^{\mathrm{STO}}$ is thus well defined by Equation (11) and the known hole concentration (= dopant concentration). At very high *p*(O_2_) (>10^3^ bar), i.e., beyond our measurement range, $c_{h}^{\mathrm{STO}}$, $c_{h}^{\mathrm{LSF}}$ and $c_{h}^{\mathrm{LSCr}}$ are constant, which translates to pinned Fermi levels for STO, LSF and LSCr that do not change with *p*(O_2_). Accordingly, the space charge potential stays constant. As soon as $c_{h}^{\mathrm{STO}}$ decreases toward lower partial pressures, $E_{F}^{\mathrm{STO}}$ rises, which is indicated by the upward arrow. For still constant $c_{h}^{\mathrm{LSCr}}$, this leads to a *p*(O_2_) dependent Δϕ with a change of 38.3 mV per decade. Such a slope is thus expected in the entire *p*(O_2_) range with accessible Δϕ and indeed the measured slope of LSCr (38.5 mV) fits this model excellently. The same is true for LSM.

**Figure 12:**
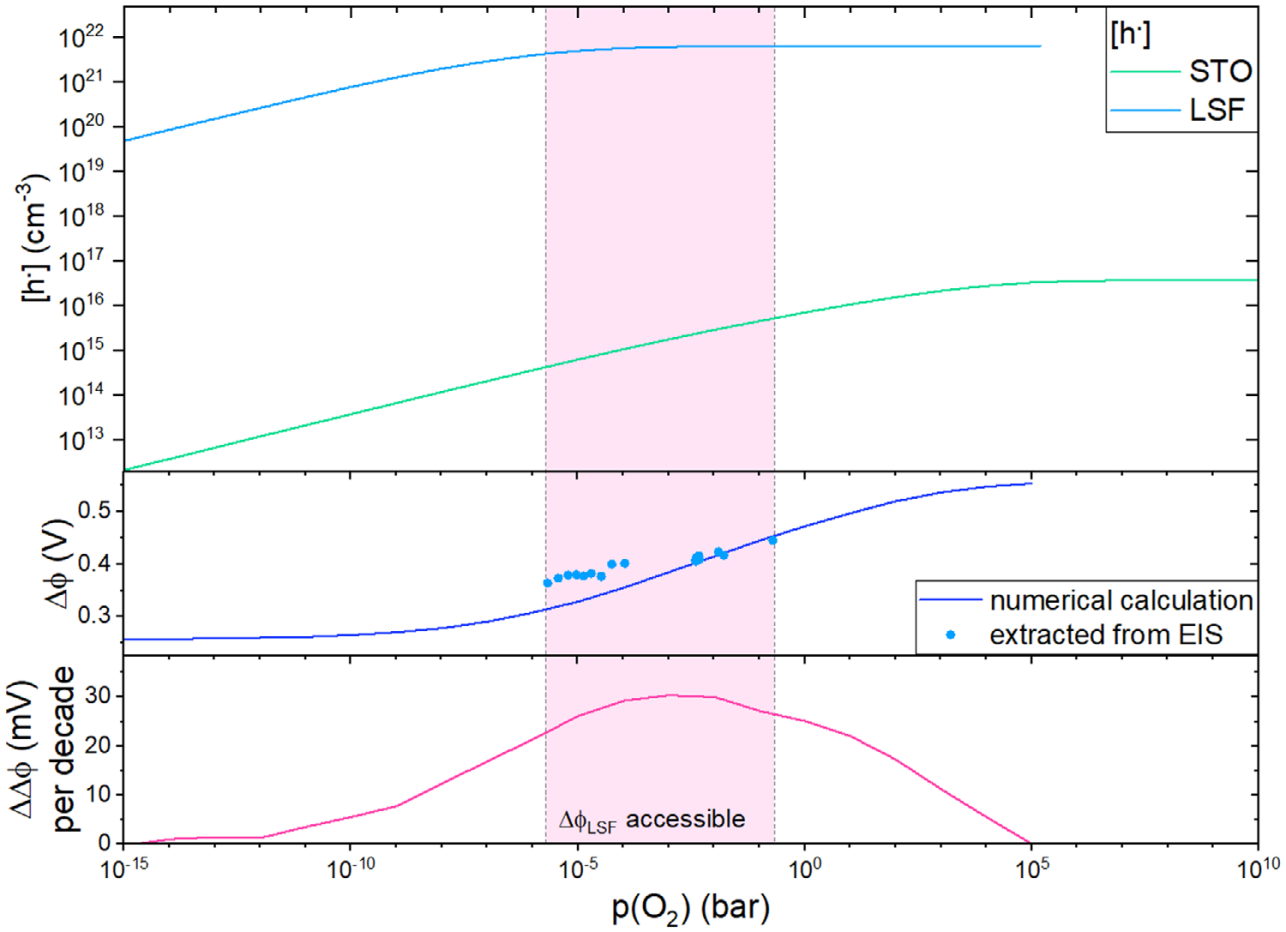
Calculated Brouwer diagrams (hole concentrations [h^.^]) for STO^[^ ^18^ ^]^ and LSF^[^ ^21^ ^]^ (top) used to calculate the change in Δϕ (bottom). The experimental value of 446 mV at 200 mbar is used as starting point. Experimental Δϕ_LSF_ values are plotted for comparison.

The situation changes for LSF. First, we consider the simplified situation of fixed slopes sketched in Figure 11. At very high pressures the situation is the same as for LSCr, with constant hole concentration and Δϕ. In the 10^3^ to 10^−6^ bar range $c_{h}^{\mathrm{STO}}$ varies and thus also Δϕ should vary accordingly. Below 10^−6^ bar, however, $c_{h}^{\mathrm{LSF}}$ strongly varies as well and a constant Δϕ is expected. Unfortunately, this final range is beyond the *p*(O_2_) regime where Δϕ could be determined for LSF. However, also the measurable slope between 1 bar and 10^−6^ bar is significantly smaller than the expected $\frac{1}{4}$ slope. This, however, is at least partly due to the fact that the *p*(O_2_) regimes in the Brouwer diagrams show broad transition ranges of roughly 5 to 6 orders of magnitude in *p*(O_2_) and for LSF we partly cover exactly those ranges. The Brouwer diagrams (obtained from data in reference^[^ ^18^, ^21^ ^]^) are shown in **Figure** **12** and these concentrations were used to predict a *p*(O_2_) dependence of Δϕ with the experimental value of Δϕ (446 mV) at 200 mbar used as starting point. The change in Δϕ calculated from Brouwer diagrams for the *p*(O_2_) range where Δϕ_LSF_ was experimentally accessible amounts to a slope between 21 and 30 mV per decade *p*(O_2_). In the partial pressure range of 10^−1^ to 10^−3^ bar experimental data is in good agreement with the predicted Δϕ change from Brouwer diagrams. At lower *p*(O_2_), Δϕ_LSF_ shows slightly higher absolute values than predicted by the model. Those deviations may be caused by difficulties in fitting the space charge arc, which only appears as a small shoulder of the STO bulk feature in impedance data. The already small ratio of *R*_SC_/*R*_STO_ becomes even smaller at low *p*(O_2_). Accordingly, a very small *R*_SC_ value has to be separated from a much larger *R*_STO_ despite strong overlap of the arcs. This might lead to flawed *R*_SC_ values and thus the deviating Δϕ_LSF_ compared to the numerical calculation. Moreover, also existence of additional *p*(O_2_) dependent interfacial core charges may effect Δϕ.

**Figure 13:**
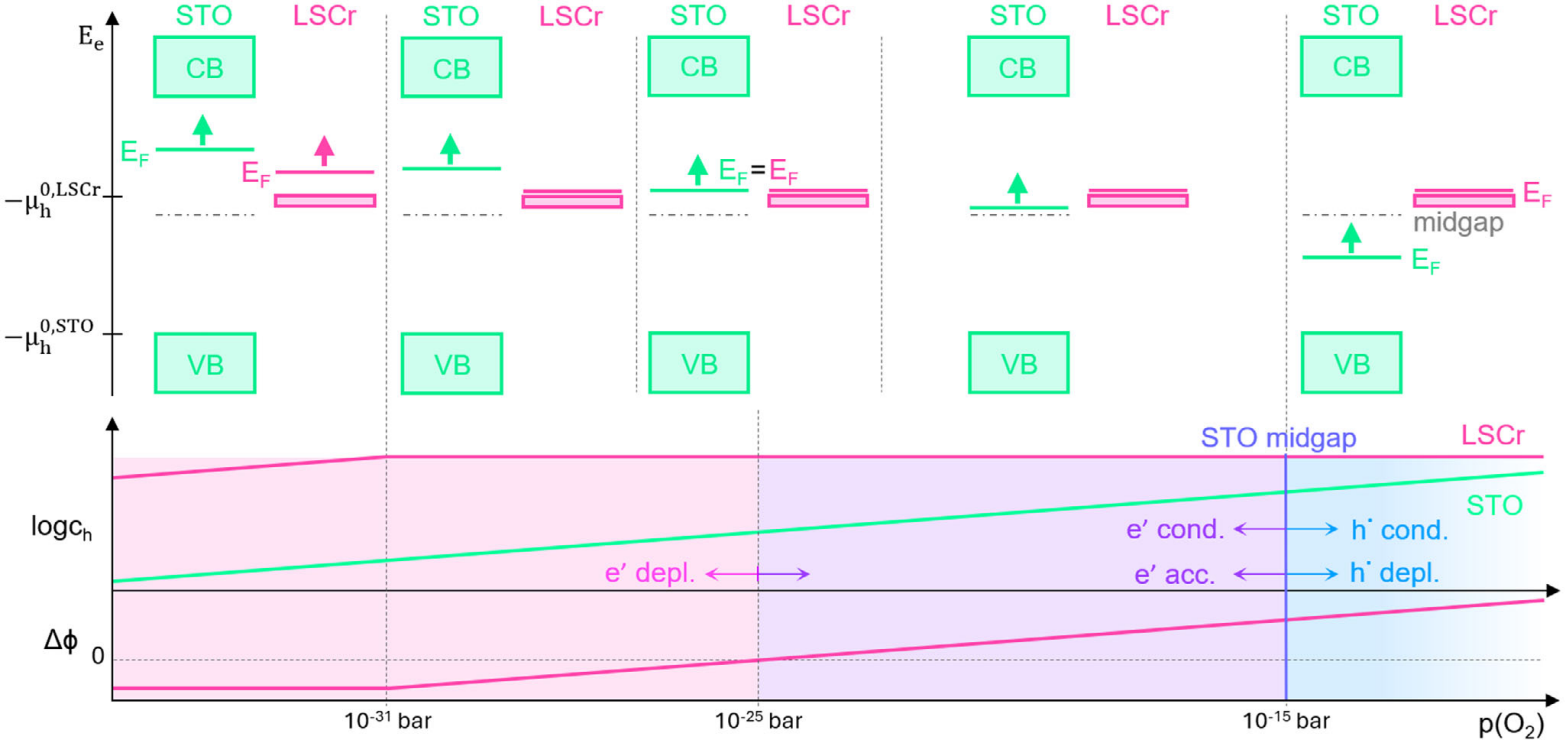
Schematic illustration of the Δϕ dependence of STO|LSCr on the *p*(O_2_) and the respective defect chemical models, specifically *c*_h_. The corresponding band schemes are given, where the Fermi level E_F_ = −µ_h_. $\mu_{h}^{0}$ equals the valence band edge for STO and represents the hole polaron level for LSCr. The width of this polaronic level is arbitrarily chosen. In dependence on the E_F_ alignments, STO can switch from hole conducting (h^.^ cond.) to electron conducting (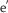 cond.) and the space charge zone in STO can be either depleted in holes (h^.^ depl.), depleted in electrons (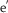 depl.) or accumulated with electrons (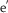 acc.). The upward arrows are plotted from the perspective of decreasing *p*(O_2_).

Based on this ability of our model to predict Δϕ, we may also extend predictions beyond the partial pressure range, where Δϕ was accessible in the given study. The following predictions refer to STO|LSCr for decreasing *p*(O_2_) and are schematically shown in **Figure** **13**. According to the defect chemical model of LSCr,^[^ ^55^ ^]^ $c_{h}^{\mathrm{LSCr}}$ is constant between 1 bar and ca. 10^−31^ bar, while $c_{h}^{\mathrm{STO}}$ decreases toward lower partial pressures. At high and medium oxygen partial pressures, changes of the space charge can be fully attributed to the change in $c_{h}^{\mathrm{STO}}$. More specific, we start with $E_{F}^{\mathrm{STO}}$ being significantly beneath midgap at high *p*(O_2_) and reach the intrinsic point of STO with equal hole and electron conduction (midgap) at ca. 10^−15^ bar. Above 10^−15^ bar, where the STO is still hole conducting, the space charge is depleted in holes. Below 10^−15^ bar, however, with $E_{F}^{\mathrm{STO}}$ being above midgap, electrons become relevant and those are accumulated in the space charge zone, as long as $\mathrm{midgap}\;\mathrm{energy}<E_{F}^{\mathrm{STO}}<E_{F}^{\mathrm{LSCr}}$. The corresponding space charge is no longer resistive but highly conductive with Δϕ > 0.

If the Fermi levels of STO and LSCr are equal ($E_{F}^{\mathrm{STO}}=E_{F}^{\mathrm{LSCr}}$), there forms no space charge and thus Δϕ = 0. For STO|LSCr this situation should be reached at ca. 10^−25^ bar. (This *p*(O_2_) is calculated from the measured space charge potential of LSCr, 901 mV at 1.5 · 10^−1^ bar and our model prediction of a decrease of 38 mV in Δϕ per decade in partial pressure, cf. Equation (12)). Since $c_{h}^{\mathrm{LSCr}}$ stays constant down to ca. 10^−31^ bar, $E_{F}^{\mathrm{STO}}$ then rises above $E_{F}^{\mathrm{LSCr}}$, causing a depletion of electrons in the STO space charge zone and a space charge potential with an opposite sign, here a negative Δϕ. From 10^−31^ bar toward even lower *p*(O_2_), $E_{F}^{\mathrm{STO}}$ and $E_{F}^{\mathrm{LSCr}}$ rise equally and thus Δϕ is expected to stay constant. Assuming a decrease of 38 mV per decade in *p*(O_2_) and using our measured Δϕ_LSCr_ at higher pressures, we can predict Δϕ ≈ −280 mV at 10^−31^ bar. Our impedance measurements were indeed performed down to 10^−32^ bar, but a space charge feature was not observed in the measured data in such reducing atmospheres. However, since already a space charge potential of 500 mV was rather difficult to measure, see LSF in Figure 5, it is unlikely that an even smaller space charge is detectable with the given experimental setup. Please note that an electron depletion (and thus a negative Δϕ) is not expected for LSF or LSM, as according to their defect chemical models, their respective Fermi levels begin to rise toward lower *p*(O_2_) (equally to $E_{F}^{\mathrm{STO}}$), as previously sketched in Figure 11 for LSF. Thus $E_{F}^{\mathrm{STO}}$ stays always below $E_{F}^{\mathrm{LSF}}$ or $E_{F}^{\mathrm{LSM}}$. These considerations indicate the broad applicability of our model. For known defect chemical data it can be used to predict space charges also in many other combinations of MIECs.

## 4 Conclusion

LSF, LSM and LSCr thin films were symmetrically deposited on STO (100) single crystal substrates using PLD and the resulting interfacial space charge regions were characterized by impedance spectroscopic measurements at 500 °C in a broad *p*(O_2_) range (1 bar to 10^−32^ bar). The oxygen partial pressure itself was determined by using STO as a conductivity based *p*(O_2_) sensor. Comparison with the Brouwer diagram of 24 ppm singly charged acceptor doped STO^[^ ^18^ ^]^ at 500 °C supported the validity of the determined oxygen partial pressures. For LSF a moderate space charge potential of 446 mV at 2 · 10^−1^ bar was found. LSM and LSCr exhibit larger space charge potentials with 814 mV at 2.2 · 10^−2^ bar and 910 mV at 1.5 · 10^−1^ bar, respectively. For all investigated MIECs a decrease of Δϕ was observed, resulting in an average slope of 13.3 mV for LSF, 37.4 mV for LSM and 38.5 mV for LSCr. Overall, Δϕ was accessible in the partial pressure range between 1 bar and ca. 1 · 10^−11^ bar for LSM and LSCr and 10^−6^ bar for LSF. At lower *p*(O_2_), space charge characterization was not possible due to an overlapping feature in the measured impedance spectra, which can be attributed to stoichiometry polarization due to ion blocking. At very low partial pressures down to 10^−30^ bar, a space feature was not observable from impedance measurements.

A model is proposed to describe and predict the dependence of the space charge potential on the oxygen partial pressure. This model is based on the assumptions, that the space charge potential can be described through either the ionic or the electronic charge carriers and focuses on the description of Δϕ through holes. Since the concentration of holes in a mixed conductor is strongly *p*(O_2_) dependent, $\frac{d\ln c_{h}}{d\ln p\left(O_{2}\right)}$ in STO and/or the MIEC is ultimately the reason for the change of Δϕ with the partial pressure. It is shown that indeed defect chemical models of STO and the MIECs, specifically *c*_h_, taken from literature can excellently predict the measured *p*(O_2_) dependencies of space charge potentials. Moreover, the model can also be used to predict complex *p*(O_2_) dependencies of interfacial space charge layers including electron accumulation or electron depletion space charge zones in STO.

## Conflict of Interest

The authors declare no conflict of interest.

## Data Availability

The data that support the findings of this study are available from the corresponding author upon reasonable request.
